# Ducklings imprint on chromatic heterogeneity

**DOI:** 10.1007/s10071-019-01273-2

**Published:** 2019-06-10

**Authors:** Antone Martinho-Truswell, Bethan McGregor, Alex Kacelnik

**Affiliations:** 1 Department of Zoology, University of Oxford, Oxford, OX1 3PS UK; 2 St Paul’s College, University of Sydney, 9 City Road, Camperdown, NSW 2050 Australia

**Keywords:** *Anas platyrhynchos*, Filial imprinting, Entropy, Innate preferences

## Abstract

Avian filial imprinting is a rapid form of learning occurring just after hatching in precocial bird species. The acquired imprint on either or both parents goes on to affect the young bird’s survival and social behaviour later in life (Bateson in Biol Rev 41:177–217, 1966). The imprinting mechanism is specialized but flexible, and causes the hatchling to develop high-fidelity recognition and attraction to any moving stimulus of suitable size seen during a predefined sensitive period. It has been observed (Martinho and Kacelnik in Science 353:286–288, 2016; Versace et al. in Anim Cogn 20:521–529, 2017) that in addition to visual and acoustic sensory inputs, imprinting may incorporate informational rules or abstract concepts. Here we report a study of mallard ducklings (*Anas platyrhynchos domesticus*) undergoing imprinting on the chromatic heterogeneity of stimuli, with a focus on how this may be transferred to novel objects. Ducklings were exposed to a series of chromatically heterogeneous or homogeneous stimuli and tested for preference between two novel stimuli, one heterogeneous and the other homogeneous. Exposure to heterogeneity significantly enhanced preference for novel heterogeneous stimuli, relative to ducklings exposed to homogeneous stimuli or unexposed controls. These findings support the view that imprinting does not rely solely on exemplars, or snapshot-like representations of visual input, but that instead young precocial animals form complex multidimensional representations of the target object, involving abstract properties, either at the time of learning, or later, through generalization from the learnt exemplars.

## Introduction

Imprinting is a specialized and rapid form of learning that occurs during an early life sensitive period. Its critical property is that brief exposure to a stimulus in the absence of any associated contingency results in a relatively stable set of affiliative behaviours towards that stimulus (Bolhuis 1991). Avian filial imprinting is a common example, the result of which can affect a bird’s whole life (Bolhuis 1991). Although occurring over the course of a few minutes (Bateson 1966) to a few days post-hatching, this learning goes on to influence much of the bird’s behaviour (primarily social preferences) (Bolhuis 1991).

Imprinting in the precocial fowl is remarkable for its speed. Just a few minutes of exposure to a moving stimulus causes a chick or duckling to recognize and respond preferentially to that stimulus in later tests (Bolhuis 1991), typically by following or maintaining proximity. In contrast to conventional associative learning, imprinting does not require a predictable sequel to the exposure, although it can be modified after more extensive experience (Bolhuis et al. 1985). The most recognizable form of this phenomenon can be witnessed in hatchlings imprinting upon their mother, whom they then follow closely for much of the first year of life. While neither chicken nor duck parents feed their young, the mother provides protection and guidance to food (Bateson 1966), enhancing the hatchlings’ chances of surviving to maturity.

The roles of visual and auditory properties of stimuli on imprinting have been extensively studied, but recent work has shown that young birds can imprint on abstract qualities of their perceptions, including relational concepts (Martinho and Kacelnik 2016). Ducklings imprinted on pairs of objects that either shared or did not share their colour or shape subsequently preferred to follow visually novel pairs of stimuli that exhibited the same relation as the previously exposed pair. This conceptual imprinting is likely to be adaptive, as a duckling must recognize its mother in spite of changes in perspective, shape, context and other transformations. The idea that avian imprinting does not depend on snapshot-like representations but on a rich set of perceived concepts has gained strength through a diversity of recent findings. For instance, chicks imprinted on objects showing partial rotation movements recognize versions of such objects rotated to novel angles, indicating sensitivity to inferred three-dimensional topography (Wood 2015; Wood and Wood 2015). The prior endowment of the imprinting system is also revealing greater than expected complexity. Chicks show innate preferences for stimuli that look hollow (Versace et al. 2016), show biologically relevant movement symmetry (Rosa-Salva et al. 2018), or move in naturalistic patterns (Miura and Matsushima 2016).

In this study, we tested whether ducklings imprinted on a high or low level of visual stimulus heterogeneity deploy that generalized imprint in subsequent testing. Generalized learning of stimulus heterogeneity (or lack thereof) is likely to be a key mechanism in many of the instances of abstract learning in juvenile birds, and is, in the natural environment, a potentially identifying signature of the mother’s visual presentation to her offspring. Though the mother may change shape or disposition at any moment, her overall level of visual heterogeneity is likely to remain relatively consistent. To test this, mallard ducklings were first exposed to visual stimuli exhibiting either heterogeneity or homogeneity of colouration and pattern, and were later tested behaviourally by giving them the opportunity to approach and follow either of two stimuli with novel colouration patterns, one homogeneous and the other heterogeneous, examining if the property of chromatic heterogeneity is itself a dimension of imprinting.

## Materials and methods

### Ethics

All procedures were carried out under the university’s animal welfare standards for vertebrates and cephalopods (University of Oxford, 2017) and approved by the Department of Zoology Ethical Review Body. No invasive procedures were carried out.

### Subjects

36 domesticated mallard ducklings (*Anas platyrhynchos domesticus*) of unknown sex were supplied by Foster’s Poultry, Gloucestershire as eggs, and returned to the supplier as young birds after participating in the experiments. The ducklings were divided into 3 groups of 12. Two of the groups were trained on (i.e. exposed to) colour heterogeneity and homogeneity, respectively, and the third group acted as control, with no exposure prior to the preference test.

### Incubation and hatching

All eggs were incubated for 28 days in a Brinsea Ova-Easy 190 incubator in fixed conditions of 37.7 °C and 40% humidity. Hatching took place in the dark to prevent ducklings forming extraneous imprints in the hatching basket. The ducklings remained in the hatching chamber for 13–24 h following hatching, as imprinting is most successful during the peak sensitive period between 13 and 40 h of age (Hess 1964).

### Priming

Before training each duckling was placed in a well-lit light priming chamber for 1 h with up to three conspecifics. This priming procedure produces improved imprinting responses in chickens (Bateson and Wainwright 1972; Lickliter and Gottlieb 1987; Lickliter and Gottlieb 1985).

### Exposure

Ducklings in the homogeneity-imprinted and heterogeneity-imprinted groups were exposed to stimuli exhibiting either colour homogeneity or heterogeneity for a total of 90 min prior to testing.

Exposure took place in three stages. First, each duckling was individually placed in an arena (1.25 × 1.25 m) with a moving training stimulus for 30 min, immediately after the completion of priming. Each training stimulus consisted of a cube 12 cm on each side constructed of foam and faced with coloured squares. The cube was suspended approximately 5 cm from the floor by invisible fishing line attached to a rotating boom. Each revolution lasted approximately 40 s, with a diameter of 1 m, as movement has been shown to enhance imprinting (Hess and Hess 1969). After the initial 30 min of training, the duckling was moved to a second training room for exposure to a second revolving cube of novel but similar hetero- or homogeneous design for a further 30 min. Training was repeated a third time, again with a novel but similarly designed stimulus. After the completion of the exposure phase, ducklings were placed in a dark chamber for a 30-min retention interval.

### Stimulus design

**Fig. 1:**
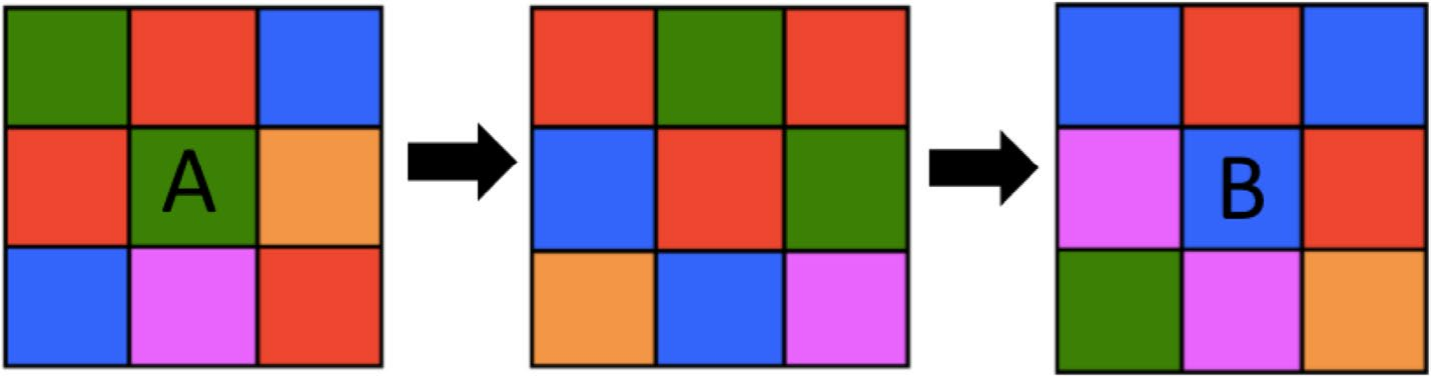
Heterogeneous stimulus production. To produce stimuli of equal heterogeneity without replicating either the colour ratios or positions of those ratios, we rotated position and then colour to produce the next presented stimulus. A stimulus exposed in training room 1 (A) would first have each coloured square change position, as in the intermediate stimulus above, then would have colours rotated through the sequence red > blue > violet > orange > green > red. This would produce the stimulus used in training room 2 (B), which would undergo the transformation again for training room 3 and the testing room

**Fig. 2:**
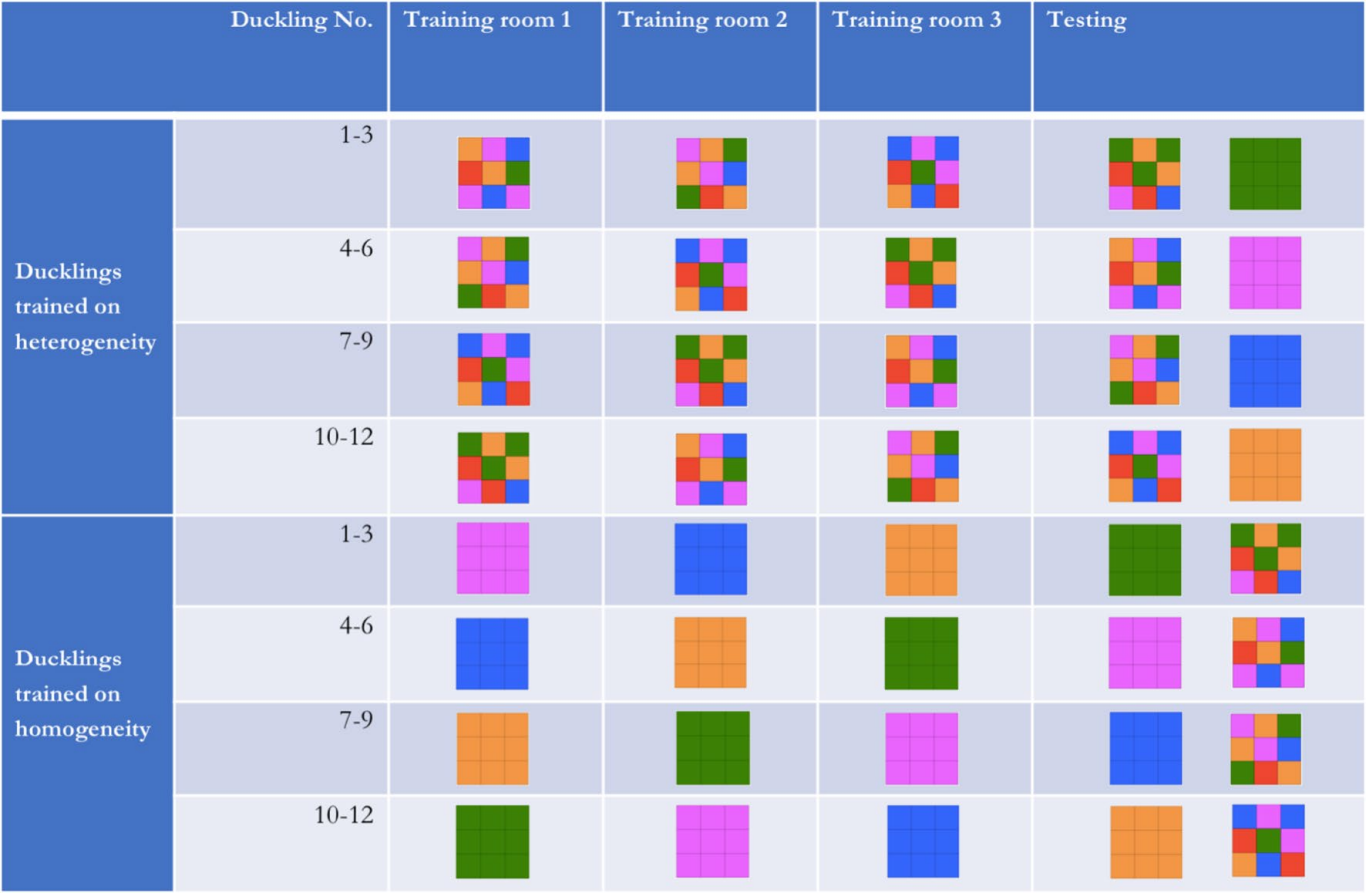
Schedule of imprinting stimuli. Ducklings were trained with three stimuli and tested with a pair of stimuli. In testing, ducklings could choose between the assigned testing stimulus against the assigned testing stimulus of the corresponding group of oppositely imprinted ducklings. Note that all stimuli used featured black lines dividing coloured cells in a grid

Each cube face displayed a 3 × 3 grid of squares (each square 4 × 4 cm). These squares were either all the same colour (homogeneous) or a random arrangement of different colours (heterogeneous), drawn from among five hues known to induce successful imprinting (Schaefer and Hess 1959; Gray 1960): green, red, orange, pink and blue. All faces of each cube displayed the same pattern. The actual stimuli used for the different duck groups and the stimulus design protocol are shown in Figs. 1 and 2. The three training cubes seen by each duckling showed different patterns but equal degree of heterogeneity. For the homogeneous cubes, this was achieved by selecting three of the five colours for all squares, so that two hues could be reserved, one of which to be used as the homogeneous alternative in the test (see Figs. 1 and 2). For the heterogeneous cube, different patterns were created through rotation of the colours across squares within each face and rotation of the colours’ frequency through a set ratio, to deal in a balanced way with the need to colour nine squares with five hues (see Fig. 1). Varying the ratio of colours was aimed at preventing ducklings from imprinting on a given colour’s total area upon the cube’s face. The use of three different designs of similar heterogeneity across the three training rooms aimed at causing imprinting on the heterogeneity of the stimuli rather than on their specific appearance. The cubes were rotated across the three training rooms and the testing room, such that four subgroups of ducklings were each imprinted on the cubes in a different order (see Fig. 2).

### Controls

The 12 ducklings used as a control group were also primed for 1 h, before proceeding directly to the dark chamber for 30 min, followed immediately by testing.

### Testing

**Fig. 3:**
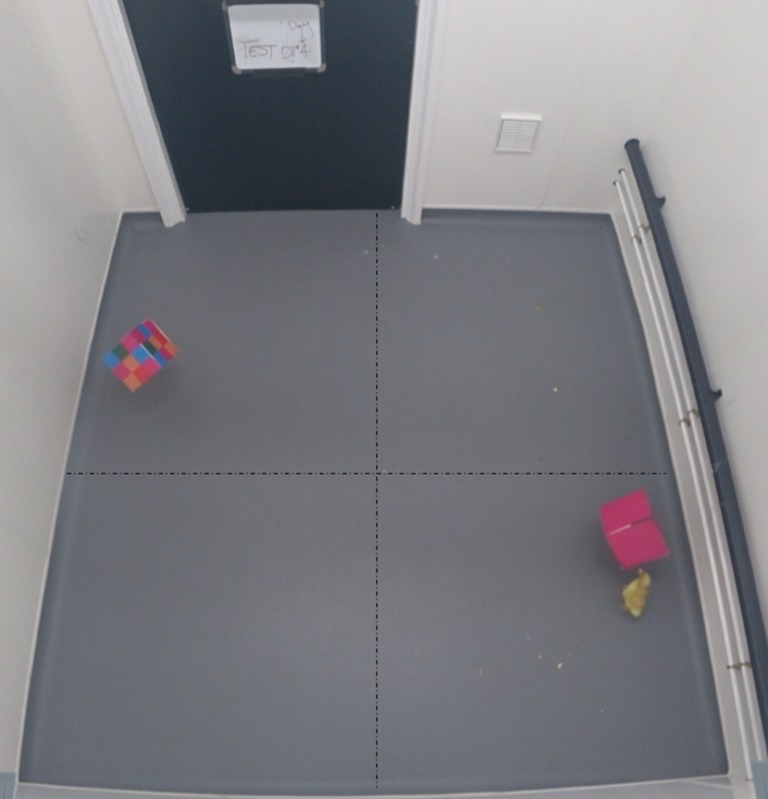
Testing chamber. The ducklings were tested in a 2 × 2 m chamber with two stimuli revolving in apposition. Here, a duckling follows the homogeneous stimulus. The notional axis superimposed over the image was used to normalize scoring across subjects. Ducklings could score one approach per stimulus per quarter of revolution

After the 30-min retention interval, each duckling completed five 2.5-min tests. Repeated consecutive tests were used to detect any change in preference over time resulting from potential re-imprinting on both testing stimuli, as testing necessarily occurred during the sensitive period for imprinting. For testing, two cubes that were novel to the subject revolved with a diameter of 1.75 m and a revolution time of 40 s about the centre of a 2 × 2 m testing chamber (Fig. 3). One of the cubes displayed a novel homogenous pattern, the other a novel heterogeneous pattern (Fig. 2). The novelty of the stimuli was achieved in the same way as that of the training cubes; through square and colour rotation. Prior to the test, the duckling was placed inside a centred start box consisting of a transparent acrylic cylinder in the darkened testing chamber. At the beginning of each test, the chamber was illuminated for 10 s before the cylinder was raised and removed, giving the duckling the opportunity to observe both stimuli before making its first approach. After 2.5 min, the lights were turned off and the duckling was returned to the start box to start a new test. The duckling was left in darkness for 30 s between tests.

### Scoring

All tests were video recorded from above using a high-definition camera. Following the method of Martinho and Kacelnik (2016), preference was measured by scoring the number of approaches the duckling made to each stimulus, with approaches defined being the direction of movement from any starting point. This scoring was completed twice, once by the experimenter and once by an independent scorer blind to subject treatment. A maximum of one “approach” to each stimulus could be scored per 90° of stimulus rotation, as per the notional axes shown in Fig. 3. This method normalized across different following styles of duckling motion, so that a duckling moving smoothly in a circle following the cube and another making many small movements could score equally (maximum of four approaches per stimulus per rotation) if they had similar bias towards each of the two stimuli. By dividing the test into discrete quadrant periods, and measuring preference in each by direction of movement, each scored movement was independent of the previous, reducing the risk of pseudoreplication of expressed preferences.

A correlation coefficient was calculated to investigate the association between the two scorers’ results. The calculated value *R* was 0.95 (*n* = 180). We used Cohen’s Kappa test to measure agreement. The coefficient *ĸ* was 0.67, which corresponds to ‘substantial agreement’ (McHugh 2012). A total of 180 tests were scored. In 143 cases (79%), the 2 scorers agreed on the duckling’s preference. In 5% of cases, the scorers assigned opposite preferences, and these tests were excluded from analysis. In cases when a scorer reported a preference and one did not (16%), a third, naïve scorer arbitrated.

## Results

Since each duckling’s preference was tested repeatedly (five times) while still in their sensitive phase, and during tests they were exposed to both kinds of stimuli, the effect of the controlled exposure should wane through each subject’s experience. For this reason, we analysed the results taking the test sequence into account.

**Fig. 4:**
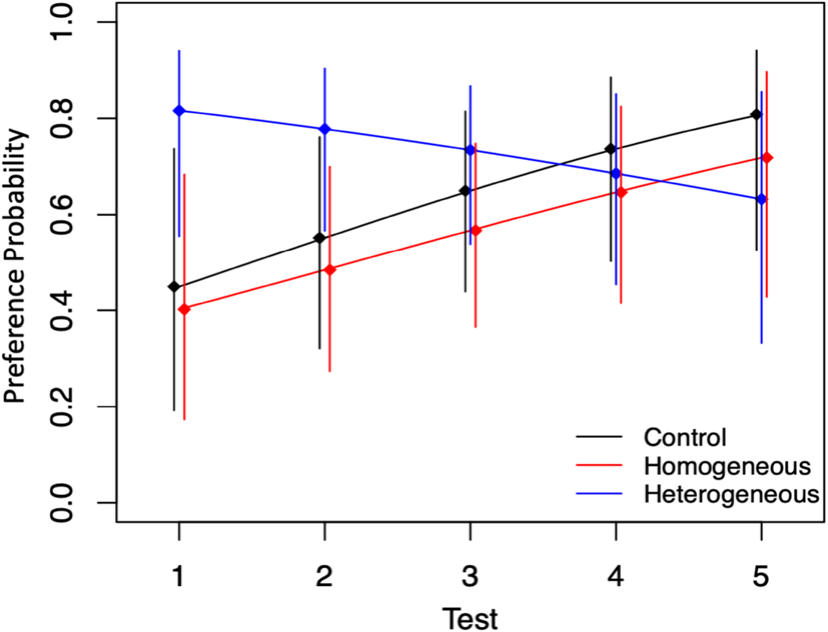
GLMM for likelihood of preference for heterogeneity. The generalized linear mixed model predicted each group’s probability of preferring heterogeneity in each test. The *y*-axis gives the likelihood of preference for the heterogeneous stimulus during testing. Ducklings in the control and homogeneously imprinted groups showed no group-level preference in the first test, and tended toward preference for heterogeneous stimuli as testing continued. Conversely, heterogeneously imprinted ducklings significantly preferred to follow heterogeneous novel stimuli in testing, but lost this preference by the fifth test

Excluding those tests in which ducklings made no movements at all or exhibited no preference (i.e. made an identical number of movements toward both stimuli), we fit a binomial penalized quasi-likelihood generalized linear mixed model (McCulloch and Neuhaus 2001) for each of the three groups across the five tests, accounting for the repeated measures across tests. The goodness of fit of the model was assessed by half-normal QQ plot which showed a good fit and no outliers (all Pearson residuals fell within a 95% envelope). The model, together with 95% confidence intervals, is shown in Fig. 4. As Fig. 4 shows, the heterogeneity-imprinted group showed significant preference for heterogeneity in the first test (its 95% CI does not include 50%, with 10 ducklings of 12 exhibiting more movements to heterogeneity than homogeneity, *p* = 0.039, two-tailed binomial test), declining towards indifference in subsequent tests, while control and homogeneity-trained ducklings showed the converse trend, although homogeneity-exposed subjects did not show a significant initial preference for homogeneity as the heterogeneity group had for heterogeneity. GLMM analysis showed that both the *y*-intercept, (preference for heterogeneity in test 1), and slope (change in preference across tests) of the heterogeneous group differed significantly from those of the control and homogenous groups (*p* = 0.045 and *p* = 0.048 for intercept and slope, respectively). The *y*-intercept for the heterogeneous group was 0.82, with 95% confidence from 0.55 to 0.94, against 0.45 (0.19–0.74) for the controls and 0.40 (0.17–0.68) for homogeneity. Binomial tests for preference in test 1 against chance were similarly significant for heterogeneity (as above) but not significant for homogeneity or the controls.

## Discussion

We found that brief repeated exposures to chromatically heterogeneous stimuli altered the preferences of young ducklings when choosing between pairs made up of two novel stimuli, one exhibiting heterogeneity similar to the original and the other being chromatically homogeneous. In contrast, exposure to homogeneous stimuli did not result in detectable differences in preferences with respect to unexposed controls. The effect was transient: preferences between the treatment groups differed when first encountering the novel stimuli, but converged after more prolonged experience with both levels of heterogeneity. This is likely due to the phenomenon of re-imprinting; as the duck undergoes its serial tests, its relative experience of the heterogeneous and homogeneous stimuli becomes, respectively, more similar. As all of the tests took place within the sensitive period, this is likely to account for the diminishing treatment effects across tests. Further, the effect was not symmetric in strength: ducks exposed to heterogeneous stimuli initially significantly preferred novel stimuli with that property, while those exposed to homogeneous stimuli or unexposed controls did not have a significant initial preference.

In spite of its rapidity and lack of obvious associative content, imprinting is known to involve behaviour of considerable cognitive complexity. In particular, it is, together with avian song learning, a uniquely suitable arena to understand the articulation of prior (‘innate’) knowhow, learning processes, and learning contents. Much is now known beyond the original notion of animals assimilating the first arbitrary visual percept and then remaining rigidly engaged to it. Both the temporal course of sensitivity to perceptual input, and the representations that guide behaviour of imprinted animals have been shown to be intricate enough to warrant multiple angles of imprinting research.

Imprinting research has shown that precocial animals display a rich repertoire of innate preferences, equivalent to priors in artificial learning systems. For instance, chicken hatchlings spontaneously prefer stimuli with biomorphic movements (Regolin et al. 2000), and hollow objects (Versace et al. 2016). The representations formed through imprinting are no less rich than the innate biases. Chickens can imprint on the numerosity of stimuli (Rugani et al. 2013), and on proto-grammatical arrangements (Versace et al. 2006). Ducklings exposed to pairs of objects that are either equal or different from each other later display a preference for pairs of novel objects with the pre-exposed relation. This is striking, because relational concept learning requires substantial reinforced training in adult vertebrates such as pigeons and baboons (Cook et al. 2003; Fagot and Parron 2010).

All of this implies that what the brain acquires through imprinting is multidimensional knowledge of objects perceived during the critical periods, and identifying the relevant dimensions is consequently an important target for experimental research. In particular, a pair of objects that are same or different also differ in the symmetry and heterogeneity of the compound. Ducklings responding to the degree of sameness may, in fact, be sensitive to the degree of symmetry or heterogeneity they perceive (Wasserman et al. 2001). In the present study, we focused on heterogeneity, a quality different but intimately related to the same/different relation between just two objects already reported in ducklings (Martinho and Kacelnik 2016). Relational concept learning through reinforcement in pigeons (Blaisdell and Cook 2005) and baboons (Fagot et al. 2001; Wasserman et al. 2001) is enhanced using heterogeneous exemplars, suggesting a role for the information entropy of stimuli independent of inter-object relations (Fagot et al. 2001; Wasserman and Young 2010).

Our data do show a learned difference between ducklings exposed to stimuli differing in degree of heterogeneity, but it is important to interpret this result cautiously since the difference is also compatible with other empirically solvable interpretations. Contrasting with the clear result with birds exposed to heterogeneous stimuli, ducklings exposed to a homogeneously coloured stimulus did not, on average, behave significantly differently from unexposed controls, and with repeated exposure to both patterns during testing they displayed a significant increase in attraction to the heterogeneous alternative, while the heterogeneity group showed a decrease. The difference in strength of preference for the pre-exposed stimulus property is likely to be caused by a higher innate sensitivity to heterogeneous candidate imprinting objects, but there is an important caveat that cannot as yet be excluded. In one respect, our homogeneous stimuli differed more starkly from each other (including between those in training and testing) than the heterogeneous exemplars. Although the frequency and positioning of the colours in the heterogenous stimuli were novel, the testing stimuli contained some instances of colours present in the training exposure, and this could have supported chromatic generalization. This latter interpretation is unlikely because in the only test in which ducklings showed a significant preference, namely those imprinted on heterogeneity in their first test, both stimuli were composed of new combinations of colours which were already familiar to the animal. A duckling relying solely on colour familiarity should thus have shown indifference in this test. Future work can examine this possibility by testing the animals whilst avoiding any repetition in colours. Finally, it is possible that, given that wild-type female mallards are chromatically heterogeneous, a preference for a specific degree of heterogeneity may be part of the innate predispositions of the newborn duckling, that is, combined and elaborated on the basis of its perceptual exposure during the sensitive period. Fortunately, these possibilities are all suitable to empirical discrimination, and further strengthen the potential role of imprinting protocols in the study of natural learning and—hopefully—the design of artificial learning systems (Versace et al. 2018).
